# Intermodal Detection of Lumbar Instability in Degenerative Spondylolisthesis is Superior to Functional Radiographs

**DOI:** 10.3389/fsurg.2022.860865

**Published:** 2022-06-06

**Authors:** Harald Krenzlin, Naureen Keric, Florian Ringel, Sven Rainer Kantelhardt

**Affiliations:** Department of Neurosurgery, University Medical Center Mainz, Mainz, Germany

**Keywords:** degenerative spondylolisthesis, slip progression, functional radiographs, Sagittal translation, MRI

## Abstract

**Purpose:**

In this study, we compare different imaging modalities to find the most sensitive and efficient way of detecting instability in lumbar spondylolisthesis.

**Methods:**

Patients presenting with spondylolisthesis from June 01, 2018 to May 31, 2020 with functional radiographs and either CT scans or MRI images were included in our single-center retrospective cohort study. The amount of translation, in millimeters, was measured on supine MRI images, CT scans, and radiographs of inclination while sitting, standing, or prone and reclination while standing using the Meyerding technique. The amount of translation was compared among the different modalities.

**Results:**

A total of 113 patients with spondylolisthesis on 125 vertebral levels were included in this study. The mean patient age was 73.52 ± 12.59 years; 69 (60.5%) patients were females. The most affected level was L4/5 (62.4%), followed by L3/4 (16%) and L5/S1 (13.6%). The average translations measured on supine CT were 4.13 ± 5.93 mm and 4.42 ± 3.49 mm on MRI (*p* = 0.3 for the difference between MRI and CT). The difference of inclination while sitting radiograph to slice imaging was 3.37 ± 3.64 mm (*p* < 0.0001), inclination while standing to slice imaging was 2.67 ± 3.03 mm (*p* < 0.0001), reclination while standing to slice imaging was 1.6 ± 3.15 mm (*p* = 0.03), and prone to slice imaging was 2.19 ± 3.02 mm (*p* = 0.03).

**Conclusion:**

We found that a single radiograph in either inclination, reclination, or prone position compared to a CT scan or an MRI image in supine position can detect instability in spondylolisthesis more efficiently than comparison of functional radiographs in any position.

## Introduction

Spondylolisthesis is derived from Greek words describing the relative displacement of one vertebra to the subjacent vertebra below. The underlying medical condition was first described by Herbinaux in 1782, and the medical term was introduced by Kilian in 1854 (1). Initially, the condition was described in obstetric literature as the displacement of the fifth lumbar vertebra against the sacrum-compromised pelvic inlet during labor. A degenerative type of lumbar spondylolisthesis was first described by Junghanns and Macnab in 1931 and 1950 (2). Vertebral dislocation mostly occurs in the anterior (anterolisthesis) or posterior (retrolisthesis) direction, while lateral displacements are rare. Two forms of spondylolisthesis are described: “isthmic” or “degenerative” (3). Degenerative spondylolisthesis develops with increasing age due to discal degeneration, increasing translational anteroposterior shear forces and concomitantly failing facet joint complexes. Isthmic spondylolisthesis appears predominantly in individuals with spondylolysis (4). The vertebral level L4/5 is most commonly affected by degenerative spondylolisthesis, while L5/S1 is the predominant site of spondylolisthesis induced by spondylolysis (5).

The natural history of spondylolisthesis involves contributory factors such as a lower intercristal line, tilting of the intervertebral disc, tropism and sagittal orientation of the facet joints, increased pelvic incidence, increased mechanical loading across the disc space, and generalized joint laxity (6, 7). While degenerative spondylolisthesis is seen as a rather stable condition, with slip progression rates of 30% over the years, spondylolysis is associated with slip progression rates of 40% and higher (8, 9). An amount of ≥3 mm difference of translation measured on different imaging modalities is defined as instability (10). More than 3 mm of translation and more than 3 mm off slip are closely related to severe clinical symptoms and are used for surgical indication (11). Episodes of back pain, radiculopathy, paresthesia, and gait disturbances are common symptoms in patients with any form of spondylolisthesis (12). Both conditions can however be aggravated by instability, causative for increased symptoms in certain positions or activities (like standing or walking).

While patients with mild symptoms might be treated with conservative measures alone, those with more severe pain or neurological deficits benefit from surgical treatment (9, 13). Cases with predominant stenosis (with or without the presence of stable spondylolisthesis) could be considered for decompression alone; instability and slip progression tilt the scales toward instrumentation and interbody fusion as treatment of choice (13). Therefore, several imaging techniques, like flexion–extension radiographs or lateral decubitus position, erect flexion, and prone traction radiographs, or the supine-prone position radiographs, have been developed to detect abnormal segmental mobility and consecutive instability (14–16). As these positions are either too complicated to standardize or hard to perform for most patients with spondylolisthesis, lateral radiographs at neutral, extension, and flexion positions are currently seen as the gold standard for detecting instability in lumbar olisthesis (17–21). Most patients receive additional CT and MRI to complete diagnostics.

We hypothesize that the comparison of lateral radiographs to CT or MRI results in a similar or superior detection rate of lumbar instability in patients with lumbar olisthesis. In this study, we investigate the feasibility to omit the combination of different functional radiographs for the detection of radiographic signs of instability to improve patient contentedness and omit inter-examiner variability.

## Material and Methods

### Study Design and Patient Population

This is a retrospective single-center study. Consecutive patients treated in our department between June 01, 2018 and May 31, 2020 with degenerative spondylolisthesis that received either CT, MRI, or both in addition to functional X-ray images were included in this study. Patients with lytic spondlosithesis and those that did not receive X-ray or any form of sectional images (MRI or CT) were excluded. Data acquisition and analysis were performed in an anonymous fashion and were approved by the corresponding ethics committee.

### Functional Radiographs

**Figure 1:**
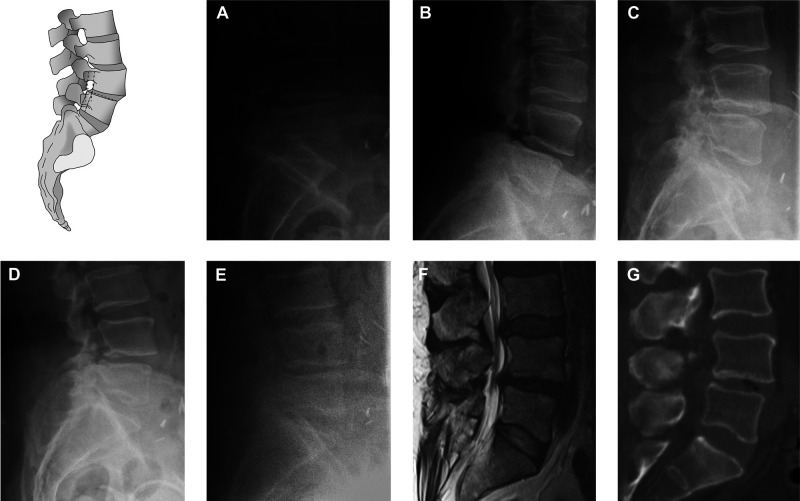
Comparisons of different imaging modalities. Sagittal translation was measured as indicated in the given pictogram. Slump siting (**A**), inclination (**B**), neutral (**C**), reclination (**D**), prone (**E**), MRI (**F**), and CT (**G**).

Standard imaging studies included X-ray standing up-right, reclination, and inclination while standing. In some cases, radiographs in slump sitting and prone positions have been added. Radiographs were acquired from digital cassettes with a focus distance of 1.15 m (Figure 1).

### Measurement of Sagittal Translation and Instability

Sagittal translation (ST) was measured on functional radiographs and CT or MRI in each level with spondylolisthesis of each patient by two independent observers. Measurements of ST performed using landmarks placed on the inferior–posterior endplate of the superior vertebra and the superior–posterior endplate of the inferior vertebra were used to calculate vertebral slippage. Instability was defined as a slip-page greater than 3 mm from the extension position. ST was measured and analyzed in absolute values. To detect segmental instability, differences in ST between all modalities for each patient were calculated. An amount of ≥3 mm difference of translation measured on different imaging modalities is defined as instability.

### Statistical Analysis

Statistical analysis was performed using Wilcoxon matched-pairs signed-rank test, student’s *t*-test (two tailed) and two-way analysis of variance (ANOVA) with Tukey’s multiple comparison post-hoc test were performed using GraphPad Prism version 8.4.2 for macOS, GraphPad Software, La Jolla, California, USA, www.graphpad.com. Inter-rater reliability was assessed using Cohen’s kappa. A value of *P* < 0.05 was accepted as statistically significant.

## Results

An overall of 113 patients (69 males; 45 females) has been included in the study. The mean age was 73.5 ± 12.6 years (age range: 41–92). In total, 125 vertebral levels with spondylolisthesis were detected and were subsequently analyzed. Patients with lytic spondylolisthesis were excluded. The highest prevalence was found in spinal levels L4/5 (61.6%) and L5/S1 (12%). All patients fulfilled the clinical criteria of instability based on the passive lumbar extension (PLE) test, the instability catch sign, or apprehension test (7) (Table 1).

**Table 1:** Demographic and radiographic characteristics of the patient sample.

	Female	Male	Total
Patients (Nb)	69 (61.1)	44 (38.9)	113
Age (SD)	73.6 (11.1)	70.6 (7.7)	72.1
Vertebral level (%)			
L1/2	1 (0.8)	1 (0.8)	2 (1.6)
L2/3	4 (3.2)	4 (3.2)	8 (6.4)
L3/4	12 (9.6)	8 (6.4)	20 (16)
L4/5	52 (41.6)	27 (21.6)	79 (63.2)
L5/S1	10 (8)	6 (4.8)	16 (12.8)
Meyerding grade (%)			
I	75 (60)	41 (32.8)	116 (92.8)
II	4 (3.2)	5 (4)	9 (7.2)
III	0 (0)	0 (0)	0 (0)
IV	0 (0)	0 (0)	0 (0)

**Figure 2:**
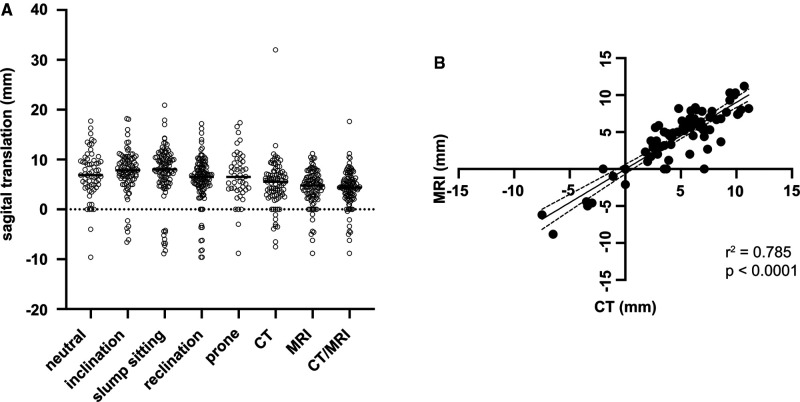
Mean sagittal translation for every imaging modality (**A**) and Correlation of sagittal translation between CT and MRI (**B**).

Mean ST while standing up-right was 7.1 ± 4.8 mm, in inclination 7.6 ± 4.3 mm, in slump sitting 7.4 ± 5.4 mm, in reclination 6.0 ± 4.6 mm, prone 6.6 ± 4.9 mm, on CT 5.1 ± 4.8 mm, and on MRI 4.4 ± 3.5 mm (Figure 2A). There was no statically significant difference between ST in CT or MRI (*p* = 0.3) (Figure 2B; Table 2).

**Table 2:** Sagittal translation.

	ST (SD)
CT	5.09 (4.78)
MRI	4.42 (3.49)
Radiographs	
Upright	7.07 (4.75)
Inclination (standing)	7.56 (4.33)
Inclination (sitting)	7.38 (5.41)
Reclination	6.01 (4.64)
Prone	6.61 (4.88)

**Figure 3:**
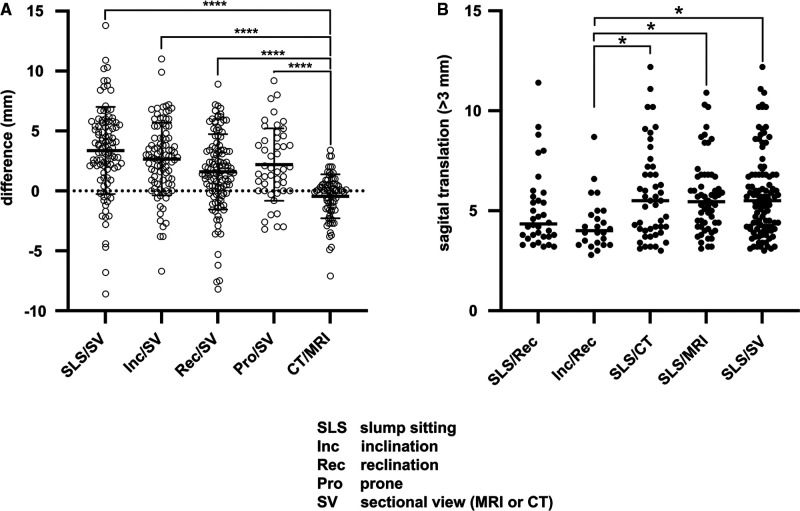
Mean difference of sagittal translation between different imaging modalities (**A**). Detection rate of pathological translation larger than 3 mm (**B**).

To determine the optimal pairing of all imaging modalities to detect segmental instability, differences in ST between modalities for each patient were calculated. As there is no significant difference between CT and MRI in ST, both modalities were subsumed as slice imaging (SI). The difference between ST on radiographs was as follows: slump sitting versus standing reclination 1.8 ± 2.5 mm (*p* = 0.29); in standing inclination to standing reclination 1.4 ± 2.5 mm (*p* = 0.19); standing neutral to standing reclination 0.1 ± 2.8 mm (*p* = 0.79); slump sitting to prone 2.5 ± 3.7 mm; and standing inclination to prone −0.5 ± 3.5 mm (*p* = 0.93). In comparison, the difference in ST between X-rays of the slump sitting radiograph to slice imaging was 3.37 ± 3.64 mm (*p* < 0.0001), standing inclination to slice imaging 2.7 ± 3.0 mm (*p* < 0.0001), standing reclination to slice imaging 1.6 ± 3.2 mm (*p* = 0.03), prone to slice imaging 2.19 ± 3.02 mm (*p* = 0.03), and standing neutral to slice imaging 6.3 ± 6.6 (*p* = 0.0013). No statistically significant difference was detected between CT and MRI; however, both were statically significant different from all radiographic modalities (*p* < 0.005) (Figure 3A).

Using functional radiographs, a total of 42 (37.17; kappa: 0.943, CI: 0.880–1.000) patients were diagnosed with segmental instability. When comparing X-rays in slump sitting with CT/MRI, 68 (56.64%; kappa: 0.873, CI: 0.781–0.964) patients show a slip progression of >3 mm (*p* = 0.02) (Figure 3B). Using lateral radiography, the sensitivity to detect slip progression was 61.11%. Comparing radiographs in slump sitting to CT or MRI, sensitivity was 80.88%.

## Discussion

Spondylolisthesis has long been an indication of spinal fusion techniques (22). Today, it is believed that merely one-third of all patients with spondylolisthesis require spinal fusion (23, 24). Controversy remains about the best imaging modalities to detect patients with increased sagittal translation due to instability, as those are believed to benefit most from spinal fusion surgery. In this study, we found that radiographs in inclination while standing or sitting compared to CT or MRI are more efficient in detecting a greater ST and higher rate of instability than the comparison of conventional series of functional radiographs. Additionally, we demonstrate that MRI is not necessary to detect segmental instability as comparable differences are also found using computed tomography.

Mean ST was significantly smaller in CT and MRI compared to each functional radiograph, while there was no difference between indicating a tendency for inadequate flexion due to pain or a sense of insecurity, which could lead to underestimation of lumbar instability (25). A similar phenomenon has been described by Morita et al., where greater flexion was acquired in functional radiographs if patients were led by hand compared to images where they were not (26). Leading patients through the motions during radiograph acquisition might help to overcome back pain and mental uneasiness about falling to a certain degree, albeit not being able to fully outweighing muscle tension counteracting segmental movement (25). This technique exposes an investigator to considerable ionizing radiation. Lying in a supine position without CT or MRI might in contrast help to relax the erector spinal muscles, leading to an improved vertebral alignment while sparing the investigator. Pieper et al. found no difference in ST between radiographs of standing–flexion and flexion–extension (27). Therefore, it was concluded that extension radiographs might not be required to evaluate lumbar instability. These reports are consistent with our findings, where inclination vs. standing and inclination vs. extension showed no statically significant differences. Cabraja et al. showed that flexion–extension radiographs compared with supine images acquired using CT revealed higher relative ST than flexion-extension radiographs alone (18). While these findings are also true in our study, one might conclude that one radiograph either in flexion, extension, or upright position compared with either CT or MRI is sufficient to detect slip progression and lumbar instability. In addition to Cabraja et al., our group did not only include patients where lumbar instability had already been evident in functional radiographs but also those without slip progression, thus strengthening the value of the accumulated data (18). In addition, we were able to show that no differences in ST exist between CT and MRI and that comparison of functional radiographs with both modalities seem better suited to detect lumbar instability than X-ray alone, as Cham et al. did previously show for MRI only.

While abnormal ST and slip progression might not be the most suited sign to select patients for surgical fusion procedures, it is still the most common diagnostic technique today. The effective radiation dose for plain radiography of the lumbar spine is thought to be 1.5 mSv, and that of a CT lumbar spine is around 4.6–7.1 mSv (28). While this might not seem much, one plain radiograph of the lumbar spine roughly equals the annual exposure to natural background radiation (29). As functional imaging often consists of multiple images taken multiple times, the effective radiation dose easily accumulates without adding diagnostic value. Especially in patients of younger age, this might be easily avoided by using a single plain radiograph together with MRI for informed decision making. In patients who are not eligible for MRI, it can be substituted by any CT available, as most patients scheduled for spinal surgery are likely to receive either CT or MRI prior to the procedure.

Taking radiation exposure, tight working hours schedules, and an already high diagnostic burden on aging patients into account, any reduction of necessary diagnostic acquisitions and simplifications of the diagnostic process are welcome additions to the daily routine. Furthermore, we were able to demonstrate that the comparison of plain radiographs to either CT scans or MRI images is more sensitive for the detection of increased sagittal translation than functional X-rays alone. As there is a multitude of different functional radiographs that lack standardized routines and depend on compliance and effort of patients and examiners alike, different results due to impaired specificity have to be expected (18, 26, 30). Simplifying the diagnostic process reduces interpatient and interexaminer differences and might help avoid misdiagnosis.

There are some limitations in this study: This is a single-center retrospective analysis not taking outcome parameters into account. It remains unclear whether lumbar instability detected by comparison of X-ray and CT/MRI is a useful parameter for surgical decision-making. Future studies will provide insight into the importance of plain film–MRI comparison for (surgical) decision making and outcome in lumbar spondylolisthesis treatment.

## Conclusion

In this study, we analyzed the efficiency of different functional radiograph modalities, CT and MRI, for the detection of instability in lumbar spondylolisthesis. We showed that a single radiograph (inclination, reclination, or prone) in comparison to CT or MRI is sufficient and even more sensitive in detecting lumbar instability. Our data advocates that putting patients through different positions during functional radiographic imaging causes avoidable radiation exposure, discomfort, and costs to our health care systems. Our observations offer a new perspective on the detection of instability in lumbar spondylolisthesis with potential positive benefits for clinicians and patients alike.

## Data Availability

The original contributions presented in the study are included in the article/supplementary material; further inquiries can be directed to the corresponding author/s.
